# The presentation, course and outcome of COVID-19 infection in people with Prader-Willi syndrome: unexpected findings from an international survey

**DOI:** 10.1186/s13023-022-02228-6

**Published:** 2022-02-21

**Authors:** J. E. Whittington, A. J. Holland, D. J. Driscoll, N. Hodebeck-Stuntebeck, A. Hoctor

**Affiliations:** 1 University of Cambridge, 18B Trumpington Road, Cambridge, CB2 8AH UK; 2 Division of Pediatric Genetics and Metabolism, Department of Pediatrics, University of Florida College of Medicine, Gainesville, FL USA; 3 Diakonische Stiftung Wittekindshof, Zur Kirche 2, 32549 Bad Oeynhausen, Germany; 4 International Prader-Willi Syndrome Organisation, Salisbury House, Station Road, Cambridge, CB1 2LA UK

**Keywords:** COVID-19, Prader-Willi syndrome, Symptoms, Outcome, Innate immunity

## Abstract

**Background:**

Prader-Willi syndrome (PWS), is a genetically determined neurodevelopmental disorder, associated with intellectual disabilities and a high incidence of obesity, diabetes mellitus, and respiratory disorders. We hypothesised that COVID-19, a viral infection which more severely affects people with these conditions, would, in people with PWS, present atypically and result in severe outcomes.

**Method:**

A structured on-line questionnaire was piloted with parents and professionals at the International Prader-Willi Syndrome Organization (IPWSO) and promoted internationally through their global network. Family members/other carers were asked to complete if someone they cared for with PWS was strongly suspected or confirmed as having COVID-19.

**Results:**

Over 1 year of the pandemic 72 responses were received, 47 adults, 25 children. The following underlying conditions were present: 16 people with PWS were overweight and 18 obese, five had diabetes mellitus and 18 sleep apnoea. Main presenting symptoms were raised temperature, fatigue/daytime sleepiness, dry cough, headache/pain, and feeling unwell, with illnesses generally lasting less than a week. Length of illness was not significantly related to age, BMI, sex, or genetic subtype. No one was ventilated or in an intensive care unit or died, one person was in hospital for four days needing oxygen.

**Conclusions:**

Contrary to our hypothesis, the PWS cohort had asymptomatic infection or mild illness. A possible explanation, supported by anecdotal evidence from parents and professional carers, is that people with PWS have a degree of innate immunity to viral infections. However, likely selection effects and a relatively low number of responses means that further evidence is needed to test this hypothesis.

**Supplementary Information:**

The online version contains supplementary material available at 10.1186/s13023-022-02228-6.

## Introduction

We report on a study of presumed or confirmed COVID-19 illness in children and adults with the rare neurodevelopmental disorder, Prader-Willi Syndrome (PWS), the most commonly diagnosed genetic cause of obesity. This syndrome has in high-resource countries an estimated birth incidence of 1:25,000 live births [1–4] and a population prevalence of 1:50,000 to 1:30,000 [1]. PWS is caused by the absence of expression of paternally inherited genes in the chromosome 15q11.2 region by one of three mechanisms: paternal chromosome 15q11.2 deletion (DEL), maternal uniparental disomy 15 (UPD) or rarely, an imprinting defect (ID) [5]. PWS may be suspected at birth due to extreme hypotonia and failure to thrive, followed later by evidence of developmental delay, relative growth and sex hormone deficiencies [6] the development of marked hyperphagia in early childhood, the presence of intellectual disabilities, and a characteristic neuropsychiatric phenotype [7].

SARS-CoV-2 (COVID-19) is a respiratory virus first described in humans living in Wuhan Province, China, as a novel cause of pneumonia. For many people, infection with the SARS-CoV-2 virus results in a relative mild illness with predominately respiratory symptoms, a dry cough and temperature, with the development of anosmia also being reported. In a brief report from Italy loss of smell and taste (anosmia and ageusia) were reported to have affected nearly 20% of those examined with COVID-19 [8]. Larsen et al. [9] used a Markov process to map the likely course of symptomatology of COVID-19 illness compared to other respiratory viral infections. They found that nausea and vomiting were also relatively early symptoms that followed the initial presentation of cough and fever. Early in the pandemic it was also reported that for older people, those who are obese, and for people with pre-existing conditions, the outcomes are likely to be more severe illness affecting other organ systems including the cardiovascular, gastrointestinal and central nervous systems and leading to a significantly increased mortality rate [10].

PWS is associated with many of the above vulnerability factors that are related to poor outcomes following a COVID-19 illness, in particular severe obesity is common if people with PWS have easy access to food and this in turn is associated with high rates of diabetes mellitus, sleep apnoea, and respiratory disorders [11]. A significant proportion of premature deaths in people with PWS are respiratory-related [12, 13]. Given their complexity of need, adults with PWS may live in shared residential settings with staff support giving rise to the possibility of an increased exposure to those viruses readily transmitted through close contact. For these reasons, there was concern at the beginning of the pandemic that people with PWS would have both an increased rate of infection and also poorer outcomes, including increased mortality, compared to the general population. Two additional concerns were, firstly, that the abnormal temperature regulation, the general absence of nausea and vomiting even when severely ill, and a high pain threshold, all observed in people with PWS, would give rise to an atypical presentation and course of a COVID-19 illness possibly leading to delayed or missed diagnoses. Secondly, that people with PWS may not appreciate the significance of symptoms or may not report their symptoms until the illness was advanced, thereby further delaying diagnosis and the use of treatments that may have had value early in the course of the disease.

This study was undertaken under the auspices of the International Prader-Willi Syndrome Organisation (IPWSO), a global charity advocating for people with PWS and their families. Forty national PWS Associations are full members and IPWSO has contacts in over 100 countries (www.ipwso.org). The aims of the study were:

- To identify the common presenting symptoms of a COVID-19illness in people with PWS and the course of symptoms over time to determine whether or not they differed from those symptoms commonly described in the typically developing populations;
- To investigate the outcomes of COVID-19 infections in people with PWS and whether or not previously identified (or other) risk factors impacted on outcomes;
- To determine whether or not any specific barriers to diagnosis and treatment had been identified by families, or others providing support, that might have affected outcomes;
- To make recommendations as to the diagnosis and management of COVID-19 illness or future coronavirus infections in people with PWS.

## Methods

A structured on-line questionnaire (see Additional file 1: Appendix) was constructed and piloted with parents and professionals at the IPWSO. Throughout the course of the pandemic the questionnaire was promoted internationally through the global network of the IPWSO, asking family members or other carers to complete if someone they cared for with PWS was strongly suspected or confirmed as having a COVID-19 illness. The data included in this analysis is that collected over a period of 12 months from the end of April 2020. For the purposes of this study the informant providing the information was considered to be the study participant and he/she consented to the study. Providing the person with PWS was well enough and agreed to this. No information was collected that would have enabled the identification of the person with PWS. In addition to information on COVID-19, the following information was requested: basic demographic information, details of living circumstances, height and weight at the time of infection, details about the diagnosis of PWS in the past and of COVID-19 now, and some additional questions about access to local health services in their country. The completed questionnaire was received by IPWSO and once anonymity was checked it was passed to JEW at the University of Cambridge. Both quantitative and qualitative data was collected with respondents being asked to include additional comments at the end of the survey and to add specific items in reply to questions if they were not included in the drop-down menus. The University of Cambridge Psychological Research Ethics Committee (reference PRE.2020.047) approved the study. The on-line survey was available in English, French, German and Spanish. The survey went live on 30th April 2020 with regular prompts sent to IPWSO’s global contacts and through the IPWSO social media. Details of the survey appear in the Additional file 1: Appendix.

### Inclusion criteria

The informant completing the on-line form was asked to confirm the following: (a) they knew the person with PWS well and have been involved in their care prior to and during the COVID-19 illness; (b), the diagnosis of PWS had been made by a doctor and if possible confirmed by a genetic test; (c) the diagnosis of COVID-19 illness was confirmed by a doctor and/or by a test or was strongly suspected; and (d) in the case of adults with PWS, if they were well enough, they did not object to the study.

## Results

Data on 72 people with PWS collected over 12 months to end of April 2021 is included. Information on the country of residence, age, gender, genetic type (where known), age and the presence or not of obesity given by the informants is summarised in Table 1 and information on pre-existing conditions and the treatments they were receiving in Table 2. The most common pre-existing conditions were mental ill-health, scoliosis and sleep apnoea.

**Table 1:** Core demographic information

Country of residence	N	Sex M/F	Genetics* Del/Dis/IC/nk	Mean age (range)	Mean BMI (range)	Number ow/ob
Argentina	2	0/2	1/1/0/0	14 (11─17)	25 (22─28)	1/0
Australia	1	0/1	1/0/0/0	22	nk	nk
Belgium	1	0/1	1/0/0/0	26	36	0/1
Canada	2	1/1	0/2/0/0	23 (12─34)	26 (26, nk)	1/0
Chile	1	1/0	0/1/0/0	21	53	0/1
France	12	9/3	4/7/0/1	27∙25 (2─51)	31 (17─50)	2/6
Germany	1	1/0	0/0/1/0	9	nk	nk
Hungary	1	0/1	0/0/0/1	41	29	1/0
Israel	2	0/2	1/1/0/0	16∙5 (15─18)	23∙5 (21─26)	1/0
Mexico	1	0/1	1/0/0/0	7	16	0/0
Netherlands	2	0/2	2/0/0/0	11∙5 (3─20)	16 (12─20)	0/0
Norway	1	0/1	0/0/0/1	6	17	0/0
Spain	2	1/1	2/0/0/0	15∙5 (15─16)	26 (19─33)	0/1
Sweden	5	4/1	2/1/0/2	32 (3─48)	24 (15─37)	0/1
Switzerland	1	0/1	0/0/0/1	36	32	0/1
United Kingdom	21	11/10	8/3/0/10	31∙86 (2─57)	29 (2─45)	5/11
United States	16	10/6	10/2/0/4	22∙31 (5─58)	27 (13─37) lePara>	5/7
Total^^^	72	38/34	33/18/1/20	25∙58 (2─58)	28 (2─53)	16/28

N = number; nk = not known; M = male; F = female; Del = deletion; Dis = disomy; IC = imprinting centre defect; BMI = body mass index; ow = overweight (BMI 25–30; children 80–95%); ob = obese (BMI > 30; children > 95%). ^^^47 adults, 25 children

*All specified genetics said to have been confirmed by a genetic test. Carers (of 5 children aged 5–8 and 6 adults, all of whom were said to have genetic confirmation) did not know genetic type. 9 carers did not know how diagnosed or genetic subtype

**Table 2:** Pre-existing conditions and treatments

Conditions**	any	diab	scol	apno	resp	Mih	card	pulm
some	57	5	21	18	6	26	4	3
Not known	1	1	1	1	1	1	1	1
None	14	66	50	53	65	45	67	68

Treatments***	any	GH	SH	Insul	diabM	apnoT	BP	psych
some	66	29	13	3	1	6	6	27
Not known	1	1	1	1	1	1	1	1
None	5	42	58	68	70	65	65	44

**Diabetes, Scoliosis, Apnoea, Respiratory, Mental ill-health, Cardiovascular, Pulmonary

***Growth hormone, Sex hormone, Insulin, Diabetic medicine, Apnoea treatment, Blood pressure medicine, Psychiatric medication

### Description of the cohort

Table 1 gives the core demographic information and country of residence of the people with PWS as provided by the family members or other informants. The reasons for low rates of obesity are dietary management, particularly by parents and PWS group homes, and that only four of the 25 children were obese and two were overweight, while 27 of 47 adults were obese and 14 were overweight despite interventions.

Median age was 25.7 years and median BMI was 28. 28 people were obese (4 were children) and 16 were overweight (2 were children).

### COVID-19 diagnosis

Fifty-six people were diagnosed by a positive test, five by a doctor but without a test, 6 because of symptoms and/or suspected from contact with tested cases and five retrospectively by antibody tests.

### Presentation and course of COVID symptoms

Twenty-one people (29.6%) had no symptoms. The most common symptoms were (Table 3): the three symptoms noted by those supporting them that the people with PWS most commonly complained about were: raised temperature 15%; fatigue/daytime sleepiness 15%; dry cough 11% and sore throat 11%. The most common three presenting symptoms observed directly by informants were: raised temperature 30%; fatigue/daytime sleepiness 20%; and dry cough 13%. These symptoms were also those reported to have most commonly persisted throughout the COVID-19 illness. Other symptoms or signs reported by the informants not included in the original survey included: fainting, feeling hot then cold, ‘fluid in ears’, rash/spots, chest wheeze, red eye, nasal congestion, breathing difficulty, and measured low oxygen saturation. No person with PWS was reported to have had peripheral oedema. We note that daytime sleepiness is a characteristic of people with PWS and so may have been over-reported.

**Table 3:** Symptoms throughout the illness

Symptom*	% of total cohort** (N)	% of those with symptoms*** (N)
Raised temperature	37 (26)	52 (26)
Fatigue/daytime sleep	32 (23)	46 (23)
Dry cough	22 (16)	32 (16)
Headache/pain	22 (16)	32 (16)
Felt unwell	22 (16)	32 (16)
Sore throat	20 (14)	28 (14)
Headache/pain	18 (13)	26 (13)
Altered bowel movement	17 (12)	24 (12)
Taste different	13 (9)	18 (9)
Increased behaviour problems	11 (8)	16 (8)

*All other symptoms occurred in less than 10% of people. **71 with data ***51 with any symptoms

### Severity of illness

‘Severe course of illness’ is defined as needed ventilation or admitted to intensive care unit or died.

Overall the severity of the illness was mild. In total six (8.5%) people were seen in hospital. One person spent four days in hospital due to low oxygen levels; one person was kept in overnight for simple monitoring; three people were seen in hospital and given oxygen then sent home; one person was given anticoagulants then sent home. Twenty-one (29.6%) people were said to be totally asymptomatic throughout. The symptom profile was reported as not changing substantially during the course of the illness other than reducing over time. Of those who visited hospital, three were from France; ages ranged from 21 to 51; five had BMI above 30.

The above quantitative data on presentation, course and outcome was complemented by information in the comment boxes of then questionnaire. Comments included the observation that others in the family had been more sick and the symptoms were similar to others but generally milder. In one case a ‘respiratory crisis’ was described leading to attendance in the Emergency Room. An asthma attack was initially diagnosed but the COVID test came back positive. The person returned home to isolate and had no subsequent complications (Table 4).

**Table 4:** Outcomes: recovery

Outcome of illnesss	Full recovery	Partial recovery	Not known
Children	19	3	3
Adults	42	4	1
Total	61	7	4
Deletion	28	2	3
Disomy	14	3	1
Total	42	5	4

As defined in the questionnaire, ‘Full recovery’ implies return to their normal self; ‘partial recovery implies remission of most symptoms but not yet back to their normal self (Table 5).

**Table 5:** Outcomes: length of illness; weight change

Length of illness	Illness 1 week	Illness 2–3 weeks	Illness.2 weeks	Not known*
Children	13	4	5	3
Adults	29	10	6	2
Total	42	14	11	5
Deletion	17	7	5	4
Disomy	13	4	1	0
Total	30	11	6	4

Weight change	Gained weight	Lost weight	No change	Not known
Children	6	0	18	1
Adults	10	10	24	3
Total	16	10	42	4
Deletion	8	4	19	2
Disomy	3	2	13	0
Total	11	6	32	2

4 asymptomatic; 1 was still symptomatic

### Relationship between risk factors (age, BMI, comorbidities) and outcome (length of illness)

There was no significant correlation between age (r = 0.024), BMI (r = 0.155), genetic subtype (r = 0.059), gender (r = -0.262) or number of comorbidities (r = 0.09) and length of illness, which are shown in the following Table 6.

**Table 6:** Relationship between risk factors (age, BMI, comorbidities) and outcome (length of illness)

	Illness < 1 week	Illness 1–2 weeks	Illness.2 weeks	Total
Meanage(sd)N	26∙6(15∙1)42	27∙4(15∙2014	25∙2(16∙9)11	26∙5(15∙2)67
MeanBMI(sd)N	29∙1(9∙7)40	26∙8(9∙8)13	25∙3(7∙9)9	28∙1(9∙4)62
N of comorbidities				
0	11	2	3	16
1	14	4	3	21
2	9	6	2	17
3	5	0	1	6
4	1	2	1	4
5+	2	0	1	3
Total	42	14	11	67
Genetic subtype				
Deletion	17	7	5	29
Disomy	13	4	1	18
IC defect	0	1	0	1
Not known	12	2	5	19
Total	42	14	11	67
Gender				
Male	19	9	8	36
Female	23	6	2	31
Total	42	14	11	67

N = number, sd = standard deviation, BMI = body mass index

### Living circumstances and lifestyle

Thirty-seven people with PWS lived with family; twenty-one in a PWS specific home; seven in a group home; four in supported living accommodation; one in hospital accommodation; and in two cases the information was missing. Two people changed residence during the pandemic. Most people (46) lived under UK-type quarantine restrictions, 9 people were confined to their place of residence, 14 lived under minor restrictions, and one person under no restrictions. In the case of 52 of the people with PWS there were known contacts with positive COVID-19 cases, in 17 no contacts were identified and in three cases the information was unknown or missing.

### Quality of care

Given concerns about whether people with PWS would receive the necessary level of care, informants were asked to describe the level of care received during the illness. Fifty-four were described as having needed no special care, 14 as receiving the best available care/similar to others, and two received limited care with concerns expressed that other people had been prioritised. The informants were asked to rate the healthcare in their country in general and for people with PWS specifically. Fifty-three informants said that their country had advanced healthcare, fifteen that their country had advanced healthcare but not always for people with PWS, two said that their country had limited healthcare, and for two the data was not available.

### Response rate

Responses were sought from parents/carers of people with PWS who actually caught COVID-19. We do not know how many were eligible, so the response rate is impossible to calculate. In the UK, we estimate, from data given in Whittington et al. [1], that there are roughly 1300–1500 people with PWS. If one in seventy was infected, our response rate would be very close to 100% for the UK. However, if one in seven was infected, our response rate would be 1%. Reasons to expect that the infection rate may have been low are given in the discussion.

Possible reasons for the low response rate are similar to those given in the discussion section on recruitment bias. Also, we depended on national PWS associations to promote the survey and this may have been difficult for their volunteer staff during lockdowns.

## Discussion

Contrary to our concerns that people with PWS might be more seriously affected than the general population due to their known risk factors, the parents/carers who responded to our survey reported generally very mild or no symptoms and the outcomes for people with PWS were positive. No one in the survey required lengthy hospital admission, intensive care treatment, or died as a result of a COVID-19 illness. The cohort included people with PWS with additional risk factors, such as obesity, and an age range up to the mid-fifties. They were not, however, a group with reported high levels of respiratory co-morbidity. Whilst this observation indicates that severe outcomes are not inevitable in this group of people, the question arises as to whether these findings can be generalised to the whole population of people with PWS, particular in countries not represented in the survey. Our aim had been to obtain responses from different countries with varying economic status and variations in access to health care; however, the reports were predominately from Europe and North America and we are not able to determine whether outcomes were different in less well-resourced countries. The survey was available in four European languages but not in other languages of relatively common usage, such as Arabic and Mandarin, or in the many minority languages. Despite our efforts our survey numbers were not as robust as we would have desired. For this and other reasons the sample cannot be considered to be representative of the global population of people with PWS who acquired COVID-19. In addition, the weaknesses of our survey were that the individuals with PWS were not tested regularly and therefore asymptomatic individuals would have been missed. However, if asymptomatic cases were missed this would not alter the fundamental proposition that people with PWS may have some innate protection against COVID-19, if anything it supports this conclusion. A number of those with PWS included did not have COVID-19 testing to prove the diagnosis and a small number of informants did not know whether those they supported had had genetic testing for PWS.

These findings could be explained by recruitment bias for a number of reasons: first, parents and other carers may have been too upset to complete the survey if the person with PWS they cared for had been severely ill or died; second, more adverse outcomes may have occurred in parts of the world where the survey was not known about, a significant language barrier existed, or there was no internet access; and third, if those providing support also had had COVID-19, their illness may have prevented them from completing the survey or affected their motivation. It is also possible that some of these cases were in fact more severe than reported but this was not obvious because of the high pain threshold and perhaps a reluctance on the part of the person with PWS to report their feelings. The first two of these possibilities are supported by unofficial reports from colleagues who heard of deaths and hospitalisations in America and South Africa but these were very much minority reports.

A very important drawback to interpretation is that this study cannot address the question as to whether or not people with PWS were more or less exposed to COVID-19. Lack of exposure may be a partial explanation for the low response rate, especially as in many countries the policy was for people with intellectual disabilities to be shielded and because of this or because of the general preference of people with PWS for lone pursuits, they may not have in fact come into long and close contact with infected people. Moreover, if exposed, the viral load may have been small for the same reasons. In a study comparing 15 fatal, 133 symptomatic and 138 asymptomatic cases of COVID-19 illnes, a high viral load was associated with more severe illness and poorer outcomes [14]. However, the qualitative data from this study would indicate that even where the whole family developed COVID-19, which is likely to have led to high levels of family exposure to the virus, the outcomes for those with PWS were still mild. Some limited support for suggesting that people with PWS might have some protection against severe disease following COVID-19 illness comes following more recent enquiries, specifically about serious illness or the deaths of people with PWS. These enquires were made close to the end of study through the global contacts of IPWSO and via the IPWSO Professional Providers and Caregivers Board (representing organisation providing care to people with PWS) to ask if they had knowledge of the deaths of people with PWS from COVID-19. Representatives from seven countries responded reporting little direct knowledge of deaths and a very small number of deaths in total and these were invariably in people with PWS who were very obese or had had very severe respiratory illness. Although in this study the number of surveys completed is small and the nature and extent of bias is difficult to quantify, when all the evidence is taken together we conclude that people with PWS are certainly not, as we had feared, more severely affected by COVID. Furthermore, it is possible that they have some protection from severe disease, except where they are extremely obese or their health is severely compromised for some other reason. This observation contrasts with some evidence from data linkage studies investigating COVID-19 illness in people with intellectual disabilities. Although findings from these studies have differed, on balance they indicate that people with intellectual disabilities are both more likely to be infected with COVID-19 and more likely to have negative outcomes, including increased mortality rates. This may be particularly the case for people with ID who have Down syndrome where a tenfold increase in mortality has been reported [15]. Such data linkage studies have the benefit of large numbers but their findings are not broken down according to the many other specific and often rare causes of a person’s intellectual disabilities. The term ‘intellectual disability’, as recorded in medical records, refers to an extremely heterogeneous group varying not only in the cause of, but also the severity of disability, and in the extent of co-morbidities. Increasingly it is recognised that, whilst people with specific genetically determined neurodevelopmental disabilities may have in common the presence of an intellectual impairment, they also differ fundamentally in their biology and the associated risks for specific physical illness. If the finding that people with PWS are protected against serious outcomes of COVID-19 illness is correct, the question arises as to how the specific genetics of PWS and the resultant phenotype might give rise to some protection and whether there are important lessons for the population as a whole.

The infection cycle of SARS-Cov-2 is well characterised. On exposure to the virus, the viral spike protein binds to the angiotensin 1-coverting enzyme 2 (ACE-2) receptors and can then enter the cell and replicate. This viral load results in an immune reaction with the release of cytokines and in some cases an enhanced pro-inflammatory response. The role of the ACE-2 receptor and the extent of the subsequent immune response appear central to understanding the risk for serious disease when exposed to the virus [16]. The fact that there was no difference in outcome depending on genetic type of PWS indicates that it is something specific relating to the core PWS genotype and the absence of expression of the maternally imprinted/paternally expressed genes located at 15q11-13 (MKRN3, NECDIN, MAGEL2, IPW, SNORD 116, SNORD 115) that results in some protection or, alternatively, such protection is a consequence of some additional aspect of the PWS phenotype, such as growth hormone and sex hormone deficiencies. This latter explanation seems unlikely as a significant proportion of the people with PWS included in the study were on replacement therapy.

Anecdotal evidence from parents of people with PWS suggests that they may have innate immunity to viral diseases because they either did not succumb to, or had a milder illness than, siblings for a range of childhood illnesses. In this survey, one respondent reported “*All three adults in the household, including the person with PWS, were sick at the same time, however, the person with PWS had the mildest case of the three of us”*. In a paper of 2010, Viordot et al. [17] concluded ‘*PWS subjects compared to adiposity-matched obese subjects demonstrate similar insulin resistance but increased low-grade inflammation. The dissociation of inflammation and central adiposity suggests that activation of innate immunity may be either a specific genetic feature of PWS or linked to the commonly associated obstructive sleep apnoea syndrome, and might offer a treatment target to reduce cardiovascular disease*.’ We suggest that our findings support the hypothesis of increased innate immunity in people with PWS.

Although people with PWS do not appear to have been severely medically affected by COVID, mental health may have suffered from constraints of lockdown while eating behaviours may have benefited from a positive effect of isolation. Somewhat opposing papers from Germany [18] and France [19] concluded: ‘*The COVID-19 pandemic has had a significant effect on the mental health of individuals with PWS, evidenced by an increase in behaviours associated with PWS, including temper outbursts, food-seeking, and irritability, which again underlines their need for specialised care. Individuals living with their families were particularly vulnerable, indicating that they and their families are in special need of support.*’ And *‘Lockdown during the COVID-19 pandemic was associated with positive effects for most French adults with PWS, with weight loss probably associated with a more favourable environment during this period. We observed no severe forms of COVID-19.*’

Finally, what are the lessons for the present pandemic or any future pandemic? Clearly, other viruses may have more, or different, adverse effects and any future pandemic would still require vigilance with respect to people with PWS. For some countries, the support of adult children as well as young children rests almost entirely with the family, so where family members are severely affected in a pandemic the support needs of the person with PWS may not be met. Where people with PWS have been at risk for severe illness related to a viral infection it would appear to be when they are very severely obese. Whilst in many parts of the world access to early diagnosis and the availability of information about the importance of controlling the food environment means that parents can prevent severe obesity and can ensure that co-morbidities are treated, this is not the case for many countries. The health care of rare disorders, such as PWS, remain a neglected area in many parts of the world [20].

## Conclusions

Contrary to our hypothesis that people with PWS would have worse outcomes from a COVID-19 illness than the general population, we found that our survey cohort had either a milder illness or an asymptomatic infection. Moreover, living with other infected people in a household or a group home, did not alter this finding. A possible explanation, supported by anecdotal evidence from parents, is that people with PWS have a degree of innate immunity to infectious illness. However, the disappointing (depending on the number of people with PWS actually infected) response rate to this survey does not enable us to make this a firm hypothesis. Further research into this possibility is needed.

## Supplementary Information

**Additional file 1.** Appendix.

## Data Availability

The dataset used in this study is available from the corresponding author (requires access to SPSS version 26 or 27).
