# Supplementary material for: The presentation, course and outcome of COVID-19 infection in people with Prader-Willi syndrome: unexpected findings from an international survey
Source: Orphanet J Rare Dis. 2022 Feb 21;17:69. doi: 10.1186/s13023-022-02228-6 (PMC8860280; doi:10.1186/s13023-022-02228-6)
Supplement: Supplementary file 1 — Additional file 1. Appendix. [file 13023_2022_2228_MOESM1_ESM.pdf]

IPWSO
International
Prader-Willi Syndrome
Organisation

COVID-19: its presentation, course and outcome in people with Prader-Willi syndrome (PWS)

This survey is being undertaken jointly by the Clinical and Scientific Advisory Board and the Professional Providers and Caregivers Board of the International Prader-Willi Syndrome Organisation (IPWSO).

What is the study about?

The aims of the survey are to determine the following:

1. How does COVID-19 infection present in people with PWS?

2. What are the course and outcomes of COVID-19 infection in people with PWS?

3. What factors influence the course and outcomes of COVID-19 infection in people with PWS?

4. What are the implications of the above for prevention and treatment in the present pandemic and any future epidemic?

Who can help and what is required?

IPWSO is asking family members, care providers of people with PWS and others who know a person with PWS who has had or may have had a COVID-19 infection to complete this form either online here or on paper (to request a pdf to print and return email office@ipwso.org). Please do NOT complete the survey if someone else has already completed it for the same person and please make a record that the survey has been completed after you have submitted. This is to avoid more than one response on the same person.

What are the possible benefits from the study?

If sufficient information is available for analysis, the findings of the survey and any recommendations will be written up and made available publicly via the IPWSO website and social media, and submitted for publication in a scientific journal. Our hope is that the findings from this survey will inform the care of people with PWS in this pandemic and also our response to future similar pandemics.

The survey

Please complete this survey after the person has come to the end of their COVID-19 infection, answering as many of the questions as you can to the best of your ability. If you are uncertain about the answers to any specific question do consult with others. The survey will take approximately 15 to 30 minutes to complete and must be completed in one go. Where a written response is required, please write in English if you feel able to or in your own language, we will arrange translation. Keep your answers short using only single words where appropriate. Thank you for your support and understanding.

Consent

The survey is anonymous. The age of the person with PWS and country of residence are requested because these may be important factors in the outcome of their COVID-19 infection. No identifiable information will be available about any individual with PWS. Taking part is entirely voluntary and if you prefer not to do so we understand. A more extensive information sheet is available on our website www.ipwso.org/covid-19 and can be printed if necessary.

If you have any questions, please contact Agnes Hoctor (office@ipwso.org). We appreciate that this survey will be particularly difficult for those family members and/or care staff who were caring for someone with PWS who subsequently passed away. If it is too difficult we understand. If there is someone else who knew the person well who you feel can help do ask them.

Thank you for your help.

Copyright © 2020 IPWSO. All rights reserved.

Respondent

Anonymous

:
Time to complete

Consent

Please indicate that you are willing to complete the survey and that you understand how your answers will be used.

1. I have understood the reasons for the survey and that I do not have to take part and I understand that by submitting this form I am consenting to taking part in the survey. *

   - Yes
   - No

2. I understand that the anonymised information provided will be used as set out in the information sheet. *

   - Yes
   - No

3. I understand that the information collected will not be used to identify the person with PWS affected by COVID-19 and all information is anonymised and will be stored securely. *

   - Yes
   - No

4. I am a family member/friend/paid carer for the person with PWS who is suspected as having or has been diagnosed as having COVID-19 infection and I have known the person concerned during the period they have been ill. *

- Yes
- No

5. If it is possible to do so, please ask the person with PWS for their assent/consent to you giving this information. Please explain that the survey is anonymous and that their identity will not be known to IPWSO. Please indicate by selecting the statement that best applies. *

- This survey has been explained to the person with PWS and they agree to the survey being completed
- The person with PWS is too ill at present to ask for their agreement but I believe that they would have agreed if they had been well enough
- The person with PWS has passed away
- Other, for example the person is a child/unable to understand the survey

The questions below apply to the person with PWS

Background about the person and information about the illness

6. Age of the person with PWS. *

7. Sex of the person with PWS (assigned or self-reported). *

- Female
- Male
- Non-binary

- [ ] Prefer not to say

8. Country of residence at time of illness. *

9. Diagnosis of PWS (select one only). *

- [ ] Diagnosis made by a doctor and confirmed by a genetic test
- [ ] Diagnosis made by a doctor but NOT confirmed by a genetic test
- [ ] Uncertain how the diagnosis was made

10. Genetic subtype (select one only). *

- [ ] Deletion
- [ ] UPD (disomy)
- [ ] Imprinting centre defect
- [ ] Other
- [ ] Not known

11. Diagnosis of COVID-19 (select one only). *

- [ ] Confirmed medically by a positive test (from a nose or mouth swab)
- [ ] Suspected and diagnosed by a doctor but not confirmed with a test
- [ ] Suspected because of known contact with a carrier and/or characteristic symptoms but not diagnosed by a doctor or by a test
- [ ] Other

12. If you replied "Other" please specify here.

13. Weight at the time of infection (please include units i.e. lb, stones or kg).

14. Height (please indicate units i.e. feet or metres).

15. Please indicate whether the person with PWS has been diagnosed with having any of the following illnesses before the onset of the COVID-19 infection (select all that apply).

- [ ] Diabetes mellitus
- [ ] Scoliosis requiring treatment at any time in the person's life
- [ ] Sleep apnoea
- [ ] Respiratory illness (e.g. asthma, chronic chest disease)
- [ ] Mental illness (e.g. psychosis or mood disorder)
- [ ] Cardiovascular disease (heart and/or blood vessel)
- [ ] Pulmonary emboli (blood clots in the leg and/or lungs)
- [ ] Other
- [ ] Not known

16. If you selected "Other" above please specify here.

17. Please indicate if the person with PWS was receiving any of the following treatments before the onset of the COVID-19 infection (select all that apply).

- [ ] Growth hormone replacement
- [ ] Sex hormone replacement
- [ ] Insulin injections for diabetes mellitus
- [ ] Tablets or injections (other than insulin) for diabetes mellitus (please list below if known)
- [ ] Treatment for sleep apnoea (e.g. CPAP at night)
- [ ] Medications for high blood pressure (please list below if known)
- [ ] Medications or treatments for mental illness, anxiety or behaviour problems
- [ ] Other
- [ ] Not known

18. If you selected “Other” above, or if you have indicated the person with PWS is on medication please list the names on the package/bottle here.

19. Please indicate whether the person with PWS had been a regular smoker of cigarettes in the past year.

- ○ Yes
- ○ No
- ○ Not known

20. Circumstances at the time of the infection (select one only).

Please indicate which of the following living circumstances best apply.

- Living at home with family members
- Living in a group setting for people with PWS, with staff support and food security
- Living in a group setting for people with and without PWS, without or with limited food security
- Living by themselves with at least daily staff support and food security
- Living by themselves with minimal support and no food security
- Other

21. If you selected "Other" above please specify here.

22. Please indicate if you noticed any of the following signs or symptoms during the COVID-19 illness (select all that apply).

- 1. Developing a temperature
- 2. Vomiting
- 3. Becoming more withdrawn or low in mood
- 4. An increase in behaviour problems compared to the previous two weeks
- 5. A decrease in behaviour problems compared to the previous two weeks
- 6. Complaining of loss of smell
- 7. Complaining that food tasted differently
- 8. Loss of appetite compared to the previous two weeks
- 9. Increase in appetite compared to the previous two weeks
- 10. Change in bowel habit (e.g. constipation, diarrhoea) compared to the previous two weeks
- 11. Complaining of feeling unwell
- 12. Complaining of pain

- 13. Becoming confused and/or disoriented
- 14. Complaining of abnormal and strange ideas (e.g. paranoid ideas)
- 15. Loss of interest in activities normally enjoyed
- 16. Sore throat
- 17. Dry cough
- 18. Intermittent sudden chest pains
- 19. Increased fatigue / daytime sleepiness
- 20. Swelling (oedema) of the legs
- 21. Other

23. If you selected "Other" in the question above please specify here.

24. Preferably by using the numbers beside the signs and symptoms above in question 22, or by typing the answers in, please list the three most significant early signs or symptoms that you observed which made you concerned that the person with PWS was becoming unwell (e.g, 1, 3, 16).

25. Preferably by using the numbers beside the signs and symptoms above in question 22, or by typing the answers in, please list the three most obvious signs or symptoms which you observed in the person with PWS during the course of the illness (e.g, 1, 2, 12).

26. Preferably by using the numbers beside the signs and symptoms above in question 22, or by typing the answers in, please list the three symptoms which the person with PWS most often reported or complained about during the course of the illness (e.g, 7, 10, 11).

27. This question is for the carers of those people with PWS who were admitted to hospital.

    What was the reason for hospital admission (select all that applied)?

- [ ] Severe breathing difficulties
- [ ] Marked deterioration in general health
- [ ] Not eating and drinking sufficiently
- [ ] The person with PWS was not in hospital (go to question 32)
- [ ] Other
- [ ] Not known

28. If you selected "Other" above please specify here.

29. If the person with PWS was in hospital which of the following treatments were given (select all that apply)?

- [ ] Admitted to the intensive care unit in the hospital
- [ ] Given oxygen to breath
- [ ] Required a ventilator to aid breathing
- [ ] Had an intravenous drip and given fluids

- [ ] Received antibiotic or antiviral medication
- [ ] Received physiotherapy to aid breathing
- [ ] Any other treatments
- [ ] Not known

30. If you selected "Any other treatments" please specify here.

31. If the person was on a ventilator in hospital please state for how many days this lasted.

32. Whether or not the person with PWS was in hospital, what was the outcome of the suspected or proven COVID-19 infection? Which of the following most accurately describe the outcome (select one only)?

- ○ Full recovery and back to their normal self
- ○ Partial recovery but not fully back to their normal self
- ○ Died as a consequence of COVID-19 infection
- ○ Died as a result of other complications secondary to COVID-19

33. Please indicate as best you can how long the person was ill with possible or diagnosed COVID-19 infection – from first symptoms to recovery or passing away.

- ○ Less than one week
- ○ More than one week but less than two weeks
- ○ More than two weeks (please specify below)

34. If you selected "More than two weeks" above please specify the length of time here.

35. For those people with PWS who passed away please answer the following to the best of your ability (otherwise please go to question 38).

What was the direct cause of death as indicated on the death certificate or told to you by a doctor?

36. Were there any other factors that were listed on the death certificate or told to you by a doctor that may have contributed to the death of the person with PWS?

37. Were there any other factors that, in your opinion, may have contributed to the death of the person with PWS?

38. Many countries in the world have placed severe restrictions on people's lives during the pandemic. Please indicate which of the following statements best describes any changes that have taken place in the area where the person with PWS lives (select the one that best applies).

- There have been no restrictions and most people are still allowed to lead their normal life
- There are relatively minor limitations - for example, not mixing with many people at a concert or at a sports event, and keeping their distance from others when out

- People must only mix with those who they share their accommodation with (for example family members or friends) and they can only leave the house if they need medical treatment, to buy essential food or there is some emergency. They may still be allowed physical activity outside the house once or twice a day.
- People must remain in their accommodation at all times and not leave unless there is an emergency. Food is delivered by the shop or bought by others to the house.

39. Have other people where the person with PWS lives or lived (e.g. family members, others with PWS) been diagnosed as having COVID-19?

- Yes
- No
- Not known

40. Has the person with PWS changed residences as a result of the COVID-19 outbreak (for example gone back to their parents' home)?

- Yes they now live in a different residence
- No they still live in the same place

41. Has the person with PWS gained/lost weight since the social isolation COVID-19 strategies commenced?

- Gained weight
- Lost weight
- Maintained the same weight
- Not known

42. Ethical aspects of care and treatment

Countries vary enormously in the health facilities that are available and in how prepared they were for the pandemic. Please make a judgement as to which statement below best describes the care the person with PWS received when they had COVID-19 (select one only).

- ○ The person with PWS had a very mild illness and could be supported where they lived
- ○ The person with PWS received the best possible care under the circumstances at the time, and similar levels of care as others with COVID-19 infection
- ○ The person with PWS received limited and poor care due to lack of resources for everybody including others without PWS with COVID-19 infection
- ○ The person with PWS received poor care because they were not seen as being a priority when health services were under such pressure

43. About the health circumstances in the country where the person with PWS was living at the time.

Please indicate which of the following best applies to the country where the person with PWS lived (select one only).

- ○ The country has advanced healthcare facilities
- ○ The country has advanced health care facilities but they are not always available to people with PWS
- ○ The country has limited health care facilities, for example limited access to doctors and to hospital services, such as intensive care
- ○ Accessing health care is very difficult because it is lacking or not available, for example, in rural settings
- ○ Other

44. If you selected "Other" above please specify here.

[ ]

45. Do you have any other comments about the person's illness, treatment and the outcome, or any other observations to report?

Thank you for your time completing this survey.

For those of you who were bereaved at this time we send our condolences and thank you particularly for helping.

46. If you would like a copy of the report sent to you please leave your email address below (your email address will only be used for the purpose of sending the report).
